# Analysis of induced components in electroencephalograms using a multiple correlation method

**DOI:** 10.1186/1475-925X-8-21

**Published:** 2009-09-24

**Authors:** Uwe Graichen, Herbert Witte, Jens Haueisen

**Affiliations:** 1 TU Ilmenau, Faculty of Computer Science and Automation, Institute of Biomedical Engineering and Informatics, Gustav-Kirchhoff-Straße 2, 98693 Ilmenau, Germany; 2 Friedrich Schiller University Jena, Institute of Medical Statistics, Computer Sciences and Documentation, Bachstraße 18, 07740 Jena, Germany

## Abstract

**Background:**

Evoked and induced activities are two typical components in the EEG and MEG time series after a stimulation. While evoked activity is phase-locked to the stimulus, induced activity is not. Present analysis methods are able to detect non-phase-locked parts of the signal, however, they do not improve the signal-to-noise ratio (SNR) of these signal components.

**Results:**

We present a new method for estimating induced activation in EEG multi-trial data sets. It is based on the multiple correlation of single trials. Our method not only detects induced components within the EEG signal, it also improves their SNR. The method is successfully tested with artificial data sets. Application to real data is exemplified using EEG data recorded in a photic driving experiment.

**Conclusion:**

We show that the SNR of the induced activity is enhanced by our method, and the method found longer lasting induced activity after the end of stimulation compared with a conventional method.

## Introduction

Time series recorded during neuropsychological experiments by means of electroencephalography (EEG) or magnetoencephalography (MEG) consist of several signal components. Activation, e.g. oscillations observed in different frequency bands, can occur spontaneously or related to a presented stimulus. Stimulus-related oscillations can be classified into evoked and induced activations [1-3]. Evoked components arise in all trials with a fixed temporal delay to the stimulus and are phase-locked. In contrast, induced activations exhibit a variance in the temporal delay with respect to the stimuli and are not phase-locked. However, induced components are often of interest, e. g. for the evaluation of neuropsychological experiments, as they refer to variable cognitive processes in the brain [4-6]. Thus, methods that can separate coexistent evoked and induced activity could enhance the understanding of information processing in the brain. EEG time series are typically very noisy and possess a low signal-to-noise ratio (SNR), rendering the detection of the individual signal components difficult. The most common approach to separating event-related activities is sample-wise averaging of the recorded trials in the time domain. Using this method, the phase-locked evoked components in the time series can be emphasized and their SNR can be improved. However, in this approach, induced components are attenuated by phase cancellation and a valuable source of information about brain activities remains unutilized.

In order to investigate induced activities, more advanced signal processing methods are required. The method of event-related synchronization (ERS) and event-related desynchronization (ERD) was introduced by Pfurtscheller et al. [7-9]. Instead of calculating the mean of the amplitude of the time series at each sample point, the instantaneous signal power is averaged to isolate the induced activities. The instantaneous signal power is calculated by squaring the amplitude at every sample point. This method was extended by Kalcher et al. [10] using the inter-trial variance calculated for all sample points. A similar approach to estimate induced activations was used by Tallon-Baudry et al. [6], Herrmann et al. [4] and Zanto et al. [11] who employed the wavelet power spectrum to calculate the instantaneous signal power. A further approach was proposed by McFarland et al. [12] who utilized a regression based subtraction procedure to estimate induced activities.

In this paper we present a new method for detecting induced components in multi-trial EEG time series. The method is based on estimating and equalizing the phase-shifts of the non-phase-locked activations in the single trials. Phase differences are calculated by the simultaneous correlation of the recorded single trials. Most notably, our approach facilitates both the detection of induced components in the signal, and the improvement of their SNR.

## Methods

The measured time series *s*(*t*) consists of the evoked, phase-locked and the induced, non-phase-locked signal components *e*(*t*) and *i*(*t*) respectively, and contains noise *n*(*t*) with an assumed expected value E(*n*_*j *_(*t*)) = 0,

(1)

An estimation of the phase-locked activity and an improvement of the SNR can be obtained by averaging over trials *s*_*j *_(*t*)

(2)

where *m *is the number of recorded trials. The latency *t *is measured with respect to some applied triggers. By computation of the average value of *m *trials, the SNR of the evoked components can be improved by . However, activities that are not phase-locked to the stimulus are also weakened by this averaging but more slowly than the noise [13].

Methods for the analysis of the induced activity commonly remove the phase-locked components from the single trials in a first step

(3)

and the instantaneous power of the remaining signal is averaged. The instantaneous power of a real-valued time series can be estimated in several ways. One common method is to sample-wise square the time series [7-10]. Another method to determine the instantaneous signal energy uses the analytic signal, calculated by way of the Hilbert transform ℋ{.} [11,14]. The complex-valued analytic signal *x*_*a*_(*t*) of a real-valued time series *x*(*t*) can be calculated by

(4)

where **i **is the imaginary unit. The induced activities in the time series trials can be estimated by

(5)

For a frequency band selective examination of the time series by these two methods, the data has to be bandpass-filtered at the beginning of the analysis.

A further method which provides additional information about the time-frequency distribution of the signal power is based on averaging the wavelet power spectra [4,9,11]. The wavelet power spectrum of a real-valued time series is the squared absolute value of the complex-valued wavelet transform  at a certain frequency *f *[15]. The induced activities are estimated by

(6)

Using the above methods phase-locked and non-phase-locked activations can be separated. However, one common drawback of these methods is that not only the instantaneous power of the induced activities but also the instantaneous power of the noise is averaged. This way, the SNR of the induced components cannot be improved.

To alleviate this problem, we propose the following multiple correlation method. First, phase differences of the non-phase-locked activations between the single time series in the investigated time segment and frequency band are estimated. A zero-phase bandpass filter BP(.) is applied to the time series  and the time segment of the data under investigation is masked using a window function WF(.),

(7)

Then, the correlation matrix *R*(*τ*_1_,..., *τ*_*m*_) of the time series 

(8)

with the coefficients of correlation

(9)

is calculated. The parameters *τ*_1_,..., *τ*_*m *_describe the temporal shift in sample points applied to the time series. Maximizing the sum of the coefficients of correlation underneath the main diagonal of *R*, one obtains a set of shift parameters (*τ*_1_,..., *τ*_*m*-1_) for which all pairs of time series correlate maximally

(10)

The time series  is used as fixed reference of the correlation.

The optimization problem is solved using the differential evolution method [16], which is a stochastic, population-based optimization algorithm. The *m - *1 parameters of the optimization problem are stored in a vector. Several of these vectors are used to compose a population of *NP *individuals  where *p *= 1,..., *NP *is the index of the individuals of the population, *g *is the corresponding generation and *n *= 1,..., *m - *1 is the index of the parameter within the individuals. The differential evolution method consists of four steps, the initialization, the mutation, the recombination and the selection. In the initialization step, the first generation of the population  is randomly created considering the domain of the parameters. In the mutation step, *NP *new vectors  with *p *= 1,..., *NP *are generated using three randomly chosen, mutually different individuals ,

(11)

*F *∈ (0, 2) is the scaling factor, which controls the amplification of the differential variation. During recombination, a population  is created by combining the elements  and  of the individuals of the population

(12)

*r *∈ [0, 1] is a random number and σ is the crossover probability, which controls the recombination. Finally, in the selection step, the fitness of the original and the newly created individuals are compared and the next generation of a population is generated

(13)

FIT(.) is the fitness function. The mutation step, the recombination step and the selection step are iterated until a termination condition is fulfilled. In the literature, a population size between *NP *= 5·*D *and *NP *= 10·*D *is recommended [16] and a population size *NP *< 2·*D *should be avoided [17], where *D *is the number of parameters of the optimization problem. In our application, a population size of 50 elements was found to be a good tradeoff between robust and correct estimation of the maximum obtained in maintainable computing time. Further, a scaling factor of 1.0 was chosen, given that we face an integer optimization problem. Note that for our application, the implementation of this algorithm in the computer algebra system Mathematica was used. We restricted the search space for the parameters *τ*_1_,..., *τ*_*m *_to integers within ± half the wave length of the lowest frequency of the non-phase-locked activations which remains in the time series . This is the lower cutoff frequency of the bandpass filter. This way, only the phase variability of the induced activation is to be determined. The time series  are corrected with estimated phase-shift parameters (*τ*_1_,..., *τ*_*m*-1_) yielding *sc*_*j*_(*t*). By averaging the time series *sc*_*j*_(*t*), one obtains an estimation of the induced activity

(14)

## Data

We applied the multiple correlation method to data sets from a previously performed EEG experiment addressing cortical activation related to flicker stimuli [18]. The experiment employed a visual stimulation using repetitive flashes of light with a certain frequency. Such stimulation can lead to an entrainment of the alpha rhythm in the visual cortex which is also called photic driving. During stimulation, the alpha frequency of most subjects is changed towards the stimulation frequency [19], and it is phase-locked. This effect is preserved shortly after offset of the stimulation [20]. Thereafter the phase-locking to stimulation frequency trails away and the alpha frequency changes back to the individual rhythm.

For proof of concept the time series of a single subject (female, age 32, individual alpha rhythm at 10.7 Hz) were analyzed. During the experiment, the subject had her eyes closed and was stimulated with a periodically flickering light. EEG (32 channels, Compumedics Neuroscan) signals were recorded simultaneously. Data were sampled at 1000 Hz and hardware filtered with a pass-band from 0.1 to 300 Hz. The alpha rhythm generally shows a large variability between subjects [21]. Therefore, the individual alpha frequency for the subject was estimated at rest condition. Then, fifteen individual stimulation frequencies were specified with fixed ratios of flicker frequency to individual alpha rhythm ranging from 0.4 to 1.6. Twenty trains of each stimulation frequency were presented to the subject. Every train comprised forty periods of flicker light. Two trains were separated by a rest period of 4 seconds.

## Validation

**Figure 1:**
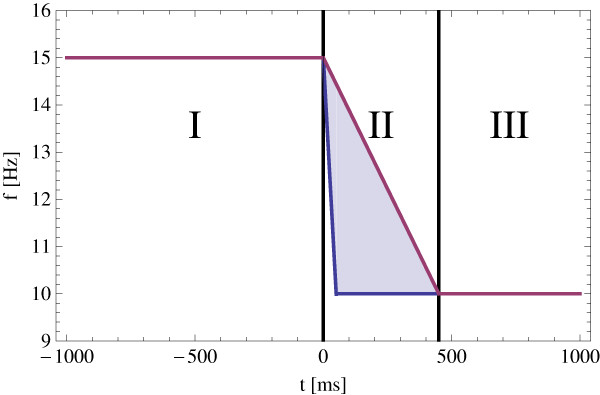
**Artificial time series**. The frequency characteristic of the artificial time series used for the validation of the presented method. It consists of three parts. The first part is a harmonic oscillation at 15 Hz. The second part is a linear chirp which starts at a frequency of 15 Hz and ends at a frequency of 10 Hz. The duration of the chirp is uniformly distributed between 50 and 450 ms. The third part is a harmonic oscillation at a frequency of 10 Hz with an uniformly distributed phasing.

Artificial time series were employed to validate the multiple correlation method. Specifically, twenty test data sets consisting of fifteen time series with 2000 samples with a sampling period of 1 ms were used. The time domain comprised -1000 ms to 999 ms. The design of the test data was chosen to simulate the behavior of the cortical activity related to flicker stimuli, recorded during the photic driving experiment. Each time series was subdivided into three sections, which correspond to the entrainment of the alpha rhythm, the change back to the alpha rhythm and the individual alpha oscillation, respectively. The first part has a duration from -1000 to 0 ms and is a harmonic oscillation at 15 Hz. The second part of the artificial time series is a linear chirp. It starts at the frequency of 15 Hz and ends at 10 Hz. The duration of the chirp is uniformly distributed in the range of 50 to 450 ms, which leads to an uniformly distributed phasing of the 10 Hz oscillation in the third part of the time series, see Figure 1. So the phase-shift of the third part of every time series in the data set is known and will be used to validate the multiple correlation method. The artificial time series were disturbed by additive Gaussian noise with a SNR of 10: 1, 5: 1, 2.5: 1, 2: 1, 1: 1, 0.5: 1 and 0.25: 1, where the following definition of SNR is used

(15)

*P*_*S *_and *P*_*N *_are the power of signal and power of noise respectively,  is the variance of noise.

For the analysis of the artificial time series, the phase-locked activities of the time series were removed first, see equation (3). Then, the multiple correlation method was applied to the time series in order to estimate the phase-shift parameters. The search space was restricted to ± 50 ms. The root mean square error (RMSE) of the known phase-shift parameters regarding the reference before and the RMSE of the remaining phase-shift after multiple correlation analysis were estimated

(16)

*t*_*j *_with *j *= 1,..., *m - *1 are the estimated phase-shifts of the time series  and *t*_ref _is the phase-shift of the reference time series .

**Figure 2:**
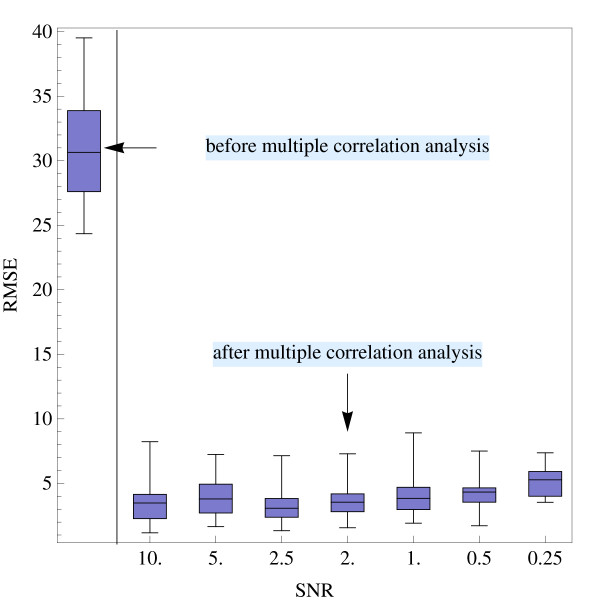
**Validation using artificial time series**. Box-and-whisker plot of the RMSE of the phase-shift parameters before (left-most item) and after applying the multiple correlation analysis

For every SNR twenty runs were performed and the mean *μ*_RMSE _and the standard deviation *σ*_RMSE _of RMSE_1_,..., RMSE_20 _of the single runs were calculated and compared with the uncorrected data, see Table 1 and Figure 2. As can be seen, by applying multiple correlation analysis to the artificial time series, the phase-shift of the non-phase-locked signal components could be considerably reduced, even for data sets with a low SNR.

**Table 1:** Results of validation using artificial time series.

	**SNR**	***μ*_RMSE_**	***σ*_RMSE_**
before multiple correlation analysis		30.57	4.03

after multiple	10: 1	3.67	1.87
correlation analysis	5: 1	3.97	1.50
	2.5: 1	3.54	1.65
	2: 1	3.81	1.59
	1: 1	4.12	1.64
	0.5: 1	4.39	1.32
	0.25: 1	5.28	1.24

Mean *μ *and standard deviation *σ *of the RMSE of the known phase-shift parameters before correction and the remaining phase-shift after multiple correlation analysis.

## Application

For the analysis of the time series, phase-locked activities were first estimated and removed from every single train, resulting in  see equations (2) and (3). The main focus of our analysis of the non-phase-locked activities was on the alpha rhythm in the time segment from 0 to 512 ms after stimulus. Therefore, before employing the correlation method, the single time series were bandpass-filtered from 2 to 20 Hz, comprising all possible stimulation frequencies. The time segment from 0 to 512 ms after stimulus offset was masked using a rectangular window. For determining the shift parameters, the search space for (*τ*_1_,..., *τ*_*m*_) was restricted to ± 50 ms. The estimated mean of the shift parameters (*τ*_1_,..., *τ*_*m*_) was  = -2.7 ms with a standard deviation *σ*_*τ *_= 35.4 ms, the minimum and the maximum were -50 ms and 50 ms, respectively, which was the border of the search space. Afterwards, the trains  were corrected by the estimated shift parameters (*τ*_1_,..., *τ*_*m*_). The resulting time series *sc*_*j*_(*t*) were averaged, see equation (14), and time frequency representations were calculated.

**Figure 3:**
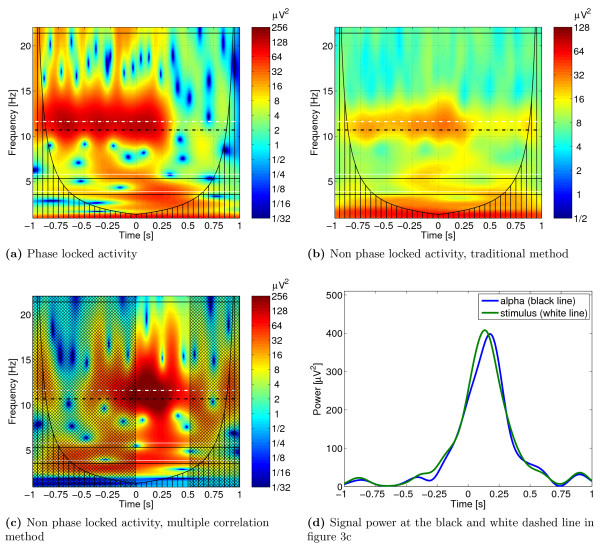
**Application to EEG data**. Time-frequency representation of the phase-locked and non-phase-locked activities in the EEG channel O1. The black dashed line marks the alpha rhythm estimated for the subject (10.7 Hz), the white dashed line the stimulation frequency (11.63 Hz). The black and white solid lines are the harmonics and sub-harmonics. The offset of the stimulation happens at *t *= 0. Figure 3a shows the phase-locked activity. Figure 3b shows the non-phase-locked activity calculated using equation (6). In Figure 3c the non-phase-locked activity estimated using the multiple correlation method is presented. The non hatched time segment is used for the correlation analysis. In Figure 3d the signal power of the oscillatory activities around the stimulation offset at the individual alpha rhythm of 10.7 Hz and at the stimulation frequency of 11.63 Hz is shown. Note the different color maps in the figures.

The adaption of the alpha rhythm to the stimulation frequency can be analyzed by estimating the phase-locked activity. Results of this part of our analysis are presented in Figure 3a. In this example, the subject was stimulated at a frequency corresponding to 1.1 times the individual alpha rhythm previously determined at rest. The adaption of the alpha rhythm to the stimulation frequency (white dashed line) can be well observed. The individual alpha rhythm estimated for the subject is marked by the black dashed line.

The synchronization of stimulation frequency and alpha rhythm continues to t = 200 ms after stimulation offset, and afterwards ends gradually. This behavior can be investigated by estimating the non-phase-locked activity after offset of the stimulation. The divergence of the synchronization of the alpha rhythm starts at t = 100 ms after stimulation offset. It is reflected in the increasing non-phase-locked activity in the time segment from 100 ms to 250 ms after stimulus offset, which slowly decreases to 350 ms. As can be seen in Figures 3b and 3c, due to the improvement of the SNR, this phenomenon can be much better recognized by our correlation procedure (Figure 3c) in comparison to the conventional procedure (Figure 3b) for the determination of induced activation.

The signal power of the oscillatory activities around the stimulation offset at the individual alpha rhythm of 10.7 Hz and at the stimulation frequency of 11.63 Hz is presented in Figure 3d. The maximum activity at the stimulation frequency appears at 134 ms after stimulation offset. In contrast, the highest activation at the individual alpha rhythm occurs 33 ms later at 177 ms after stimulation offset.

## Discussion

We have presented a new method for the detection of non-phase-locked components in EEG time series. In contrast to traditional procedures, our novel method estimates and compensates for the phase-shift of the induced components in the investigated single trials. This way, it is possible to isolate induced components in EEG time series and, moreover, to simultaneously improve their SNR.

Latency adjustment averaging techniques related to the introduced multiple correlation method were already used to estimate evoked potentials [22]. In this method a template of the expected evoked potential is fitted to the single time trials. A traditional stimulus-locked evoked potential is used as template and cross-correlation is applied as similarity measure. Single trials which do not pass a criterion level are excluded from further analysis. In the next step the single trials are realigned at the latency point which correlates best to the template, and the evoked potentials are estimated by these latency adjusted trials. This method can not be applied to detect induced activity, however. After removing the phase-locked components from the single trials they have zero mean. A template can thus not be determined. In our multiple correlation method a criterion similar to the criterion level in the latency adjustment averaging technique [22] could be established. Trials with an estimated shift parameter on the border of the search space could be treated as noise and excluded from the averaging.

The application of the multiple correlation method requires *a priori *information about the frequency band and about the time segment where non-phase-locked activity is expected in the investigated time series. Both parameters, the chosen frequency band and the time segment, also influence the optimization step which is employed to estimate the phase-shifts of the trials. The search space for the optimization can be constrained depending on the chosen frequency band. If a certain frequency is investigated, the search should be restricted to ± half of its wavelength. For investigating a frequency band, ± half the wavelength of the lower cutoff frequency should be used. The length of the analyzed time segment should be greater than or equal to the wavelength of the observed frequency. On the other hand, it should be short enough to keep the phase constant in the time segment of each trial.

Most notably, the SNR of the estimated non-phase-locked signal components can be improved simultaneously by  similar to the estimation of evoked activities. This could not be achieved by traditional methods so far.

For a comprehensive analysis of the common EEG frequency bands, the time series can be bandpass-filtered. Afterwards, the multiple correlation method can be applied to the separated frequency bands.

Note that the computational costs of the procedure increase exponentially with the number of examined trials. Therefore, the procedure is suitable only for the evaluation of experiments with few time series. The optimization step of the method is particularly time-consuming. Endeavors to reduce the computing time should thus focus on this part of the procedure. One approach could be the use of evolution strategies [23]. At present the multiple correlation method is programed in a sequential manner. Some steps, e. g. the calculation of the correlation matrix and the optimization step, offer the potential for parallelization which again could decrease computation time.

## Competing interests

The authors declare that they have no competing interests.

## Authors' contributions

HW and JH supervised the project, analyzed the final results, and helped drafting and revising the manuscript. UG developed the presented method, validated and applied it, analyzed the results and drafted the manuscript.
